# Intraoperative Diagnosis of Situs Inversus Totalis in Laparoscopic Cholecystectomy: Taking Into Consideration Patient-Centered Care in a Limited-Resource Case

**DOI:** 10.7759/cureus.78501

**Published:** 2025-02-04

**Authors:** Ximena Camila Vázquez-Guerra, Jorge Arath Rosales-Isais, Victor Ilich Hernandez-Garza, Juan Manuel Valdivia-Balderas, Luis Adrian Alvarez-Lozada, Alejandro Quiroga-Garza

**Affiliations:** 1 Human Anatomy Department, Clinical-Surgical Research Group (GICQx), Universidad Autonoma de Nuevo Leon, School of Medicine, Monterrey, MEX; 2 General Surgery Division, Instituto Mexicano del Seguro Social, Monterrey, MEX; 3 Human Anatomy Department, Clinical-Surgical Research Group (GICQx), Universidad Autónoma de Nuevo León, School of Medicine, Monterrey, MEX

**Keywords:** acute calculus cholecystitis, hepatobiliary anatomy, intraoperative finding, laporoscopic cholecystectomy, patient-centered care, situs inversus abdominals

## Abstract

Situs inversus (SI) is a rare congenital disorder in which the abdominal and thoracic organs create a mirror image orientation of their typical anatomical positions. In this article, we present the case of a 49-year-old female individual who underwent a scheduled laparoscopic cholecystectomy (LC) due to postprandial colic pain in the epigastrium. A prior ultrasound examination, conducted a year earlier, revealed the presence of small gallstones within the gallbladder, and a follow-up preoperative ultrasound suggested the possibility of SI, though the diagnosis was ruled out by a third ultrasound. Standard LC was commenced with umbilical trocar placement; however, SI was confirmed, and trocar placement was mirrored to the left side. Accurate preoperative diagnosis is crucial for surgical planning and to ensure patient safety. Performing an LC on a patient with SI poses unique challenges and requires heightened surgical skills and adaptability by the operating surgeon with extensive anatomical knowledge to mitigate risks and optimize patient outcomes.

## Introduction

Situs inversus (SI) is a rare congenital disorder characterized by the mirror-image transposition of the abdominal and thoracic organs [1]. This condition has a reported incidence of 1:10,000 to 1:20,000 [2]. Due to its rarity, it presents significant challenges in both diagnostic and therapeutic procedures [2]. Therefore, it is essential that radiologists, surgeons, and other healthcare specialists be well-informed and understand anatomical variants to effectively manage such complex cases [3]. The first laparoscopic cholecystectomy (LC) in a patient with SI was reported in 1991, and since then, 101 more successful cases have been documented [4].

Managing cholecystitis in the context of SI is particularly challenging. The diagnosis may be delayed due to symptoms manifesting contralaterally to those of a typical case [5,6]. Accurate diagnosis through imaging studies requires radiologists or radiology technicians to be familiar with SI, as early suspicion is crucial. Diagnosis can be achieved by ultrasound or X-ray imaging, which can confirm the total situs inversus. Computed tomography (CT) and magnetic resonance can provide greater diagnostic precision [7].

In cases where a diagnosis cannot be made preoperatively, the surgeon must adapt intraoperatively to this complex scenario. The surgical team may need to be repositioned to perform the procedure in a mirror-image orientation due to the transposition of the abdominal and thoracic organs, including the gallbladder, which can consequently increase the operating time [8]. In such cases, it is imperative that the surgeon possesses extensive anatomical knowledge, substantial visuospatial awareness, and surgical expertise to minimize the risk of complications [9,10].

We present a case of an LC in a patient with SI totalis that was not confirmed preoperatively. We discuss the preoperative decision-making process, intraoperative challenges encountered, and the existing evidence. This case is reported in accordance with the SCARE guidelines [8].

## Case presentation

A 49-year-old female individual with no significant medical history, aside from a previous cesarean section, was scheduled for an LC. The patient had a body mass index of 35.2 (obese) and presented with repetitive postprandial colic pain in the epigastrium. A prior ultrasound examination conducted more than a year earlier identified the presence of small gallstones within the gallbladder, without further pathological or abnormal findings. A subsequent ultrasound suggested a "possible" SI of the gallbladder, prompting the need for a third ultrasound, which ruled out SI. Notably, all three ultrasound examinations were conducted by ultrasonographers (non-medical personnel with ultrasound training) at another institution, and only the study reports were available for review. Due to the patient’s limited financial resources and increasing frequency of biliary colics, no additional imaging studies (CT or MRI), were requested by the surgeon. The patient was counseled on the diagnostic uncertainty and the potential for unexpected intraoperative findings. She consented to proceed with the surgery to avoid further delays and additional expenses. The preoperative assessment did not give rise to any other anatomical variation. All laboratories were within normal range.

In light of the findings from the second ultrasound, the patient was positioned with both arms tucked to accommodate potential changes in laparoscopic monitor positioning and rearrangement of the surgical team. During anesthesia protocol, cardiac monitoring suggested dextrocardia, and the electrodes were repositioned to align with the heart's reversed orientation. Under general anesthesia, standard LC was commenced, with the surgeon on the left of the patient. An 11 mm trocar was placed at the umbilicus using the Hasson technique to establish pneumoperitoneum, and a 30° 10 mm telescope was used for visualization.

**Figure 1:**
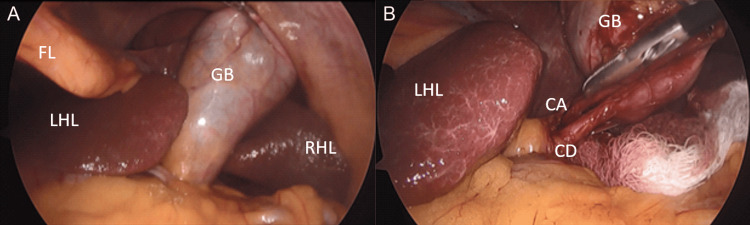
Laparoscopic view of situs inversus of the liver and gallbladder. (A) Initial view of the gallbladder following traction of the fundus. (B) Critical view of safety during cholecystectomy, demonstrating only the cystic duct and cystic artery entering the gallbladder. FL: falciform ligament; GB: gallbladder; LHL: left hepatic lobe; RHL: right hepatic lobe; CA: cystic artery; CD: cystic duct.

During the laparoscopic diagnostic examination, the stomach was observed on the right side of the abdomen, the liver with the gallbladder on the left, and the heartbeat was visualized most prominently on the right hemidiaphragm. The surgeons were repositioned to the right side of the patient, and the monitor was placed contralaterally. Under direct visualization, one 11 mm trocar was inserted in the epigastrium and one 5 mm trocar in the left hypochondrium. Adequate traction and dissection of the gallbladder were achieved, obtaining a clear critical safety view of the cystic duct and artery before placing clips and cutting (Figure 1). The liver bed was carefully dissected, and the gallbladder was successfully released and extracted. Hemostasis and textile count were confirmed. All trocars were removed under direct visualization. The procedure was classified as a Nassar II cholecystitis. Both the procedure and postoperative recovery were uneventful. The patient was discharged the following day and managed on an outpatient basis. At the two-week follow-up, the patient had recovered well with no complications reported.

## Discussion

A total of 101 laparoscopic cholecystectomies (LC) in patients with situs inversus (SI) were documented in the medical literature up to 2022 [4]. In these studies, no complications were reported, only an increase in intraoperative time. Despite this, standardized surgical protocol has yet to be established for managing such cases [6]. These procedures typically require a surgeon possessing substantial experience and comprehensive anatomical knowledge to perform the procedure effectively and ensure patient safety.

Surgeons must recognize their limitations, particularly in cases of SI, where mirror-image anatomy demands extensive anatomical knowledge and enhanced visuospatial skills [11-14]. In the case presented, the operating surgeon maintained a critical safety view and successfully proceeded without complications, despite the mirror-image anatomy. However, this highlights the necessity of proper prior training, including recognizing and localizing anatomical structures, to ensure safe and effective management in such atypical cases [8].

A preoperative diagnosis significantly influences the planned surgical approach, patient positioning, and surgeon’s readiness and preparation [15]. In this case, the suspicion of SI prompted the surgeon to prepare for potential changes in the surgical approach by positioning the patient with both arms tucked in. This facilitated the movement and repositioning of the surgical team and the laparoscopic equipment, allowing for a seamless adaptation to the unexpected mirror-image anatomy. However, the lack of a definitive preoperative diagnosis represented a significant limitation that should have been addressed.

Evidence indicates radiologists demonstrate greater accuracy than ultrasonographers in identifying anatomical structures such as the gallbladder. The diagnosis of SI is most effectively achieved through a multidisciplinary approach, with the radiologist playing a critical role in diagnostic accuracy and minimizing the risk of misinterpretation [16,17]. However, financial constraints limited the patient’s options, as she had already undergone three ultrasounds. Adding advanced imaging modalities, such as CT or MRI, would have further delayed the surgery, without altering the therapeutic approach. Additionally, given the patient’s history of recurrent biliary colic, delaying surgery would have increased the risk of complications. Notably, current evidence suggests that SI does not elevate the incidence of anatomical variants beyond mirror-image anatomy or predispose to additional complications [7].

Patient-centered care emphasizes the importance of respecting patients’ preferences, needs, and values, ensuring these principles guide clinical decision-making while avoiding unnecessary delays to deliver timely and effective care [18]. In this case, the patient was thoroughly counseled on the diagnostic uncertainty and potential intraoperative findings, ultimately providing informed consent to proceed with surgery to mitigate further financial burden and delays. Preoperative confirmation of SI would not have altered the planned course of treatment, as LC would remain the procedure of choice, performed by the same surgical team.

While adherence to established protocols is crucial, exceptional cases such as this highlight the need to balance standardized guidelines with patient-specific considerations, thereby ensuring timely, ethical, and individualized care.

## Conclusions

Preoperative diagnosis of SI totalis is essential for surgical planning, but this case demonstrates that intraoperative identification requires the surgeon's expertise and adaptability to ensure successful outcomes. It highlights the importance of balancing surgical proficiency with patient-centered care, addressing ethical and practical challenges by prioritizing patient preferences, timely care, and resource limitations. Tailoring care to individual circumstances is crucial for delivering effective, ethical, and timely interventions in complex clinical scenarios.

## References

[1] Peeters H, Devriendt K (2006). Human laterality disorders. Eur J Med Genet.

[2] Eitler K, Bibok A, Telkes G (2022). Situs inversus totalis: a clinical review. Int J Gen Med.

[3] Tapia-Nañez M, Quiroga-Garza A, Guerrero-Mendivil FD (2022). A review of the importance of research in anatomy, an evidence-based science. Eur J Anat.

[4] Enciu O, Toma EA, Tulin A, Georgescu DE, Miron A (2022). Look beyond the mirror: laparoscopic cholecystectomy in situs inversus totalis-a systematic review and meta-analysis (and report of new technique). Diagnostics (Basel).

[5] Salama IA, Abdullah MH, Houseni M (2013). Laparoscopic cholecystectomy in situs inversus totalis: feasibility and review of literature. Int J Surg Case Rep.

[6] Takei HT, Maxwell JG, Clancy TV, Tinsley EA (1992). Laparoscopic cholecystectomy in situs inversus totalis. J Laparoendosc Surg.

[7] Fernández-Reyes BA, Flores-González AK, Alvarez-Lozada LA (2022). The importance of simulation training in surgical sciences. Int Surg J.

[8] Sohrabi C, Mathew G, Maria N, Kerwan A, Franchi T, Agha RA (2023). The SCARE 2023 guideline: updating consensus Surgical CAse REport (SCARE) guidelines. Int J Surg.

[9] Strasberg SM, Brunt LM (2017). The critical view of safety: why it is not the only method of ductal identification within the standard of care in laparoscopic cholecystectomy. Ann Surg.

[10] Reitano E, de'Angelis N, Schembari E, Carrà MC, Francone E, Gentilli S, La Greca G (2021). Learning curve for laparoscopic cholecystectomy has not been defined: a systematic review. ANZ J Surg.

[11] Terho P, Sallinen V, Leppäniemi A, Mentula P (2020). Does the surgeon's caseload affect the outcome in laparoscopic cholecystectomy for acute cholecystitis?. Surg Laparosc Endosc Percutan Tech.

[12] Quiroga-Garza A, Teran-Garza R, Elizondo-Omaña RE, Guzmán-López S (2020). The use of clinical reasoning skills in the setting of uncertainty: a case of trial femoral head migration. Anat Sci Educ.

[13] Moulton CA, Regehr G, Lingard L, Merritt C, MacRae H (2010). Slowing down to stay out of trouble in the operating room: remaining attentive in automaticity. Acad Med.

[14] Dawkins A, George N, Ganesh H (2017). Radiologist and sonographer interpretation discrepancies for biliary sonographic findings: our experience. Ultrasound Q.

[15] Turner EN, Fisher KL (2016). Situs inversus with liver metastases: diagnosis with sonography and computed tomography. J Diagn Med Sonogr.

[16] Chaouch MA, Jerraya H, Dougaz MW, Nouira R, Dziri C (2021). A systematic review of laparoscopic cholecystectomy in situs inversus. J Invest Surg.

[17] Machado NO, Chopra P (2006). Laparoscopic cholecystectomy in a patient with situs inversus totalis: feasibility and technical difficulties. JSLS.

[18] Narayan AK, Miles RC, Milton A (2023). Fostering Patient-centered equitable care in radiology: AJR Expert panel narrative review. AJR Am J Roentgenol.

